# Re-pairing DNA: binding of a ruthenium phi complex to a double mismatch†

**DOI:** 10.1039/d4sc01448k

**Published:** 2024-05-16

**Authors:** Tayler D. Prieto Otoya, Kane T. McQuaid, Neil G. Paterson, David J. Cardin, Andrew Kellett, Christine J. Cardin

**Affiliations:** a Department of Chemistry, University of Reading Whiteknights Reading, RG6 6AD UK c.j.cardin@reading.ac.uk; b Diamond Light Source Ltd Harwell Science and Innovation Campus Didcot Oxfordshire OX11 0DE UK; c SSPC, The Science Foundation Ireland Research Centre for Pharmaceuticals, School of Chemical Sciences, Dublin City University Glasnevin Dublin 9 Ireland

## Abstract

We report a crystal structure at atomic resolution (0.9 Å) of a ruthenium complex bound to a consecutive DNA double mismatch, which results in a TA basepair with flipped out thymine, together with the formation of an adenine bulge. The structure shows a form of metalloinsertion interaction of the Λ-[Ru(phen)_2_phi]^2+^ (phi = 9,10-phenanthrenediimine) complex at the bulge site. The metal complex interacts with the DNA *via* the major groove, where specific interactions between the adenines of the DNA and the phen ligands of the complex are formed. One Δ-[Ru(phen)_2_phi]^2+^ complex interacts *via* the minor groove, which shows sandwiching of its phi ligand between the phi ligands of the other two ruthenium complexes, and no interaction of its phen ligands with DNA. To our knowledge, this binding model represents a new form of metalloinsertion in showing major rather than minor groove insertion.

## Abstract 2

The complex [Ru(phen)_2_phi]^2+^ creates a bulge site when crystallised with a DNA dodecamer which includes the Pribnow box sequence.

## Graphical abstract

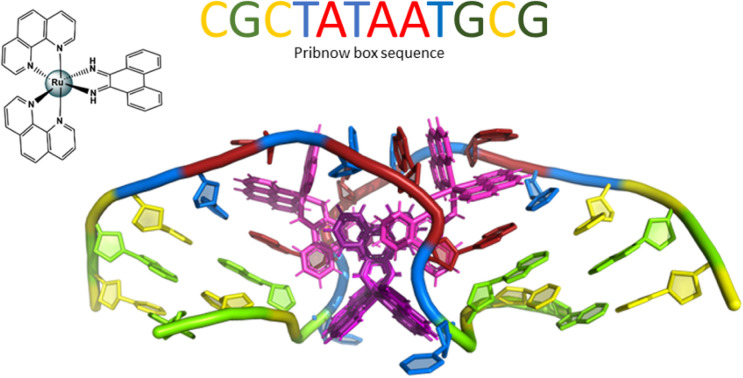

## Introduction

**Fig. 1:**
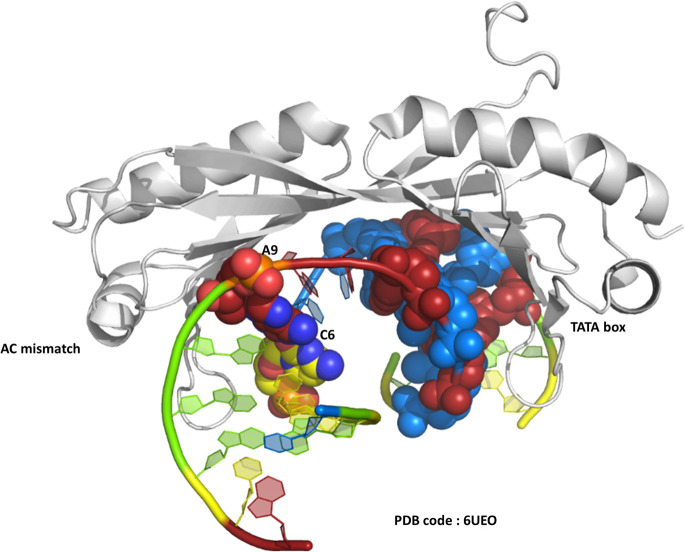
The TATA-box binding protein, bound to both the TATA-box DNA sequence and an A–C mismatch base pair. Protein shown as grey ribbon. DNA bases use the conventional colour scheme of adenine – red, thymine – blue, guanine – green, cytosine – yellow. The TATA box residues are shown in spacefill mode, and the NAKB colour scheme is used, unless stated, throughout.

One of the original observations about the first B-DNA double helix model was that there were three C–G hydrogen bonds but only two A–T interactions.^1^ Since then, the greater flexibility of AT-rich tracts of DNA has been seen in many contexts, such as in the structure of the TATA box binding protein (TBP),^2^ a transcription factor responsible for binding specific sequences next to genes known as promotor regions, where the large bend of 80° induced by the protein is possible due to recognition in the minor groove. The recognition of the TATA box by binding proteins was first established by X-ray crystallography in the 1990s, and there are now over 30 sets of coordinates available.^2–4^ In a recent example, from 2019, a mismatched base pair AC was shown to facilitate binding of TBP (Fig. 1).^5^ In this case, recognition is through curvature of the minor groove, and there is a mismatch three base pairs remote from the TATA/TATA sequence recognised by TBP.

**Fig. 2:**
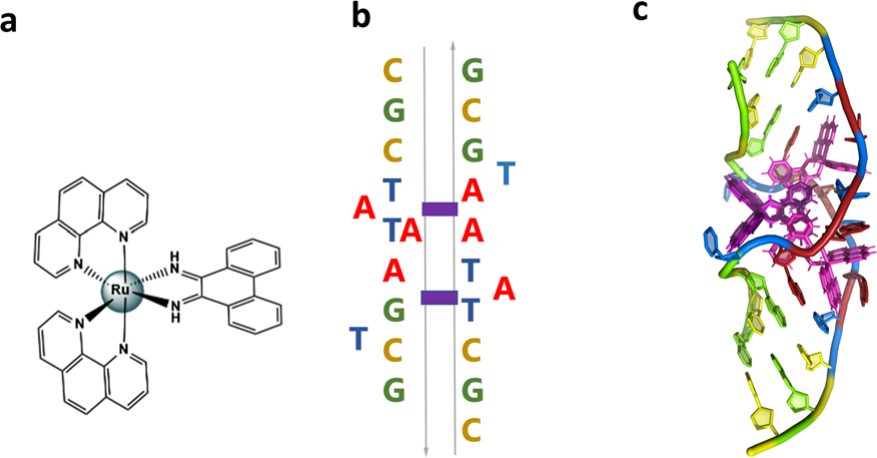
(a) Structural formula of Λ-[Ru(phen)_2_phi]^2+^; (b) schematic showing the re-pairing of the bases in the reported structure. The purple blocks highlight the binding sites of the complex. (c) Image showing the large DNA bending. The overall assembly, characterised by a twofold rotational symmetry. Each asymmetric unit is made up of a DNA single strand binding a Λ-[Ru(phen)_2_phi]^2+^ with occupancy 1 and a Δ-[Ru(phen)_2_phi]^2+^ with occupancy 0.5. The ruthenium complexes are shown in purple.

We have recently published a detailed X-ray crystallographic and solution study of the binding of the ruthenium complex Λ-[Ru(phen)_2_phi]^2+^ (Fig. 2a) to B-DNA, showing, for the first time, sequence selective intercalation from both grooves.^6^ Symmetrical major groove intercalation was seen at the central TA/TA step of the d(CCGGTACCGG) sequence used, whereas angled minor groove intercalation, stabilised by an imine-sugar hydrogen bond was seen at the adjacent GG/CC steps. The extensive solution binding studies examined a wide range of sequences, and most of the data can be explained in terms of the structural model presented in this study. Unexplained, however, is the remarkable stabilisation of a TATA containing sequence by Λ-[Ru(phen)_2_phi]^2+^, the more noteworthy because it is enantiospecific, seen only for the lambda enantiomer. The annealed duplex, d(GCTTTATAAAGC)_2_, gives a +22.4 °C increase in UV thermal melting temperature compared to the untreated control (Δ*T*_m_) with Λ-[Ru(phen)_2_phi]^2+^, but only +5.8 °C with Δ-[Ru(phen)_2_phi]^2+^. The work presented here suggests a possible interpretation of that striking result. Furthermore, our results position Λ-[Ru(phen)_2_phi]^2+^, a major groove binder, as an ideal candidate for the development of modified-triplex forming oligonucleotides as inhibitors of gene expression. Related complexes have recently been shown to have useful antitumour properties and to be useful building blocks for specific targeting.^7,8^

Non-complementary base pairs or mismatches can occur due to a range of factors such as replication errors,^9^ misincorporations^10^ and cytosine methylation,^11^ at a frequency of 1 per 10^9^–10^10^ base pairs per cell division,^12^ and these alter the natural interaction between base pairs. There are eight non-Watson and Crick alternatives or mismatches, which include the purine-pyrimidine G : T and A : C, the purine–purine G : G, A : A and G : A pairing, and the pyrimidine–pyrimidine C : C, T : T and C : T, and unlike the canonical base pairs, their properties depend on their nearest neighbour configuration.^13^ The mismatches in adjacent positions are less studied; there are very few experimental and no structural studies concerning consecutive double mismatches.^14^ Mismatches can occur in eukaryotic and/or prokaryotic DNA, such as the Pribnow box consensus sequence. The Pribnow box consensus sequence is the sequence 5′-TATAAT-3′ of six nucleotides that is an essential part of a promoter site of DNA – located at the −10 position upstream of the bacterial transcription start site for transcription to occur in prokaryotes.^15,16^ Structural studies of this relevant sequence are important for the design of therapeutic agents; this is why much effort was put into crystallising this historically intractable, biologically relevant DNA motif.^17^

DNA bending is believed to be a key feature by which base mismatches or base insertions/deletions are recognised; one example being the highly bent DNA structure found in the complex with MutS and MutSα, enzymes responsible for the first echelon of post replication mismatch repair.^18–20^ Although the role of such DNA features is not fully understood in the mismatch repair pathway, it is hypothesised that the mismatch repair protein initially binds non-specifically to DNA, and then probes for increases in local flexibility in the DNA due to the presence of the mismatch. From potential energy, or “free-energy of mean force (PMF)” profiles associated with DNA bending, it has been demonstrated that the bending of either homoduplex or heteroduplex DNA is not a spontaneous process.^21,22^ Therefore, small molecules capable of binding these unusual base pairs are important tools for therapeutic and fundamental research. Understanding atomic details of the structure of such small molecule/DNA complexes can help to uncover their specific binding mechanisms, and can open up new opportunities for structure-based drug design to target specific disease-related DNA structure.^21^ Also if the combination results in novel structural distortions and in synergistic effects *in vitro* and *in vivo*, resistance may be overcome by either drug alone. Significantly, DNA bending in the absence of MutS has been found to be rather difficult to describe correctly.

In the present work we report the crystal structure of the Pribnow box consensus sequence d(CGCTATAATGCG) when mispaired in the presence of *rac*-[Ru(phen)_2_phi]^2+^.^23–27^ The non-complementary Pribnow box sequence 5′-TATAAT-3′ is incorporated into a modification of the classic Dickerson–Drew dodecamer self-complementary sequence d(GCGGAATTCGCG).^28,29^ The resulting assembly contains both enantiomers of the complex, whose synthesis and DNA binding properties were reported many years ago.^23^ The rhodium analogue of the complex, [Rh(phen)_2_phi]^3+^, has been known for many years to photocleave DNA on irradiation, later explored with the extended ligand chrysi (=5,6 chrysenequinone diimine) for mismatch detection in duplex DNA.^30^ The authors tried and failed to obtain structural evidence for the binding modes of the rhodium analogue, but accumulated a large amount of useful data.^23–25,27^ We used the ruthenium analogue as our main aim was to obtain structural data, not to carry out photocleavage experiments. Therefore we worked with a photoinactive complex to ensure that the crystallographic experiment would not be affected by photodamage. The crystal structure presented here shows bending of the DNA at the mismatch point after metalloinsertion by the ruthenium complexes. The DNA bends towards the minor groove with widening of the major groove.

## Results and discussion

### Crystallisation and data collection

The sequence d(GCTTTATAAAGC) and several other AT-rich well-matched sequences gave no crystals with [Ru(phen)_2_phi]^2+^, either as the racemic mixture or with resolved enantiomers.^6^ The DNA sequences used were synthesised and purified by Eurogentec, and the pure ruthenium enantiomers, as the chloride salts, were prepared and characterised as previously described.^6^ Crystallisation is in general a somewhat unpredictable process when the binding mode is intercalation, and we would expect to test a range of sequences in the course of a project such as this. In the previous work, we used 10 different sequences before we were successful, which is fairly typical of the success rate. With the doubly mismatched 
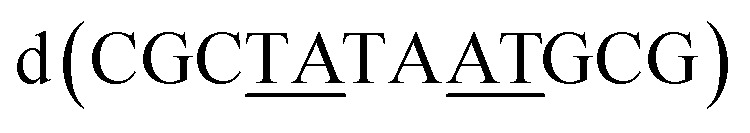
 (Fig. 2b), however, very well diffracting red crystals formed from the racemic mixture. DNA crystals containing the complex [Ru(phen)_2_phi]^2+^ cations (both enantiomers) were obtained by vapour diffusion (Fig. 2c) (from sitting drops at 277 K) from a 0.4 μL drop containing 250 μM d(CGCTATAATGCG), 250 μM [Ru(phen)_2_phi]^2+^Cl_2_, 0.01 M MgCl_2_ hexahydrate, 0.05 M HEPES sodium, and 4 mM lithium chloride. Crystallisation experiments were performed using commercially available screens (NATRIX 1 and NATRIX 2, Hampton Research). X-ray data were collected on beamline I03 at Diamond Light Source. DIALS was used to scale the data through the xia2 pipeline, with the FAST_EP protocol used to derive the initial SAD map into which the starting model was built, including the phasing, carried out using the SHELXC/D/E pipeline^31^ using the SAD method. The built structure was refined using the program REFMAC5,^32^ with the restraints for the metal complexes created using ELBOW from the PHENIX package. Manual model editing and rebuilding was done using the standalone program COOT.^33^ 4.9% of reflections were used for the *R*_free_ test. The model gave a final *R*_work_/*R*_free_ of 0.1359/0.1594. Final coordinates and data were deposited as PDB code 8CMM. Data collection and summary refinement statistics are shown in Table S1.†

### Description of the structure

**Fig. 3:**
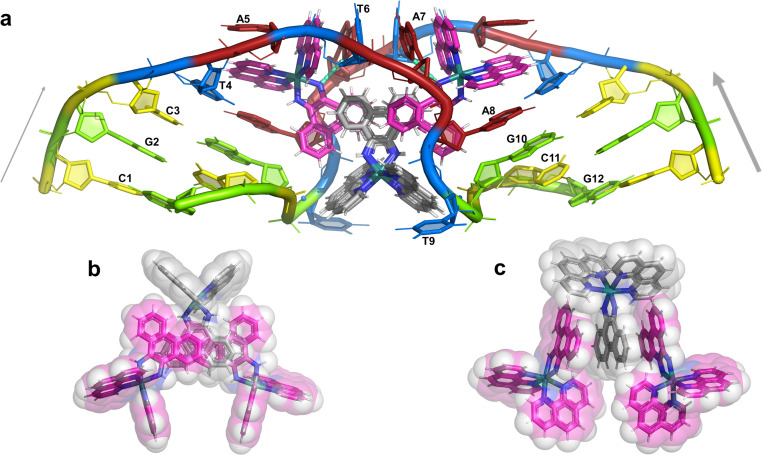
The complete assembly (a) the bases of one chain are numbered, with the chain direction indicated. Base T9 is flipped out, and base A5 is ‘bulged’, central base pairs are T4–A8 and T6–A7. The Λ-complex (magenta) binding is stabilised by a hydrogen bond to A7, shown in cyan. The Δ-complex (grey) stacks between the two Λ-complexes, through the phi ligands; (b) and (c) two views showing the stacking of the complexes in the crystal, shown as sticks and semi-transparent spheres, respectively.

The overall structure, determined to 0.9 Å resolution, is that of a severely kinked duplex, shown in Fig. 3a. The asymmetric unit of the crystal structure is composed of one d(CGCTATAATGCG) strand, one Λ-[Ru(phen)_2_phi]^2+^ cation and one Δ-[Ru(phen)_2_phi]^2+^ cation at 0.5 occupancy, along with 69 fully occupied water sites (peaks visible in the 2Fo–Fc map at 1.5*σ* = 0.88 e Å^−3^). There are also 2 potassium ions (assigned using electron density criteria), and a lithium ion (assigned using geometric criteria). There is some residual disorder at C11 and G12, but otherwise the structure is very well ordered. A central twofold rotation axis relates the two halves of the assembly. At each end there are three CG base pairs, with the central TATAAT sequence binding the two Λ-complexes and showing a modified base pairing pattern to accommodate the mismatches. The T4 base pairs with A8 on the opposite strand, so that T9 is flipped out. This shift allows T6 to pair with A7 on the opposite strand. The Λ-[Ru(phen)_2_phi]^2+^ complex is bound between the three adenine bases, A5 from one strand, and A6 and A7 from the opposing strand, generating the 80° kink observed at this step, and with a stabilising hydrogen bond between A7 and one imino –NH of the metal complex. The overall effect of the kinking is to open up the major groove and compress the minor groove. The stacking of the enantiomers of the metal complex is shown in two views as Fig. 3b and c. The crystal packing, illustrating the solvent channels, is shown in Fig. S1,† and a section of the map, illustrating the excellent quality of the data, as Fig. S2.†

### The Λ-[Ru(phen)_2_phi]^2+^ binding site

**Fig. 4:**
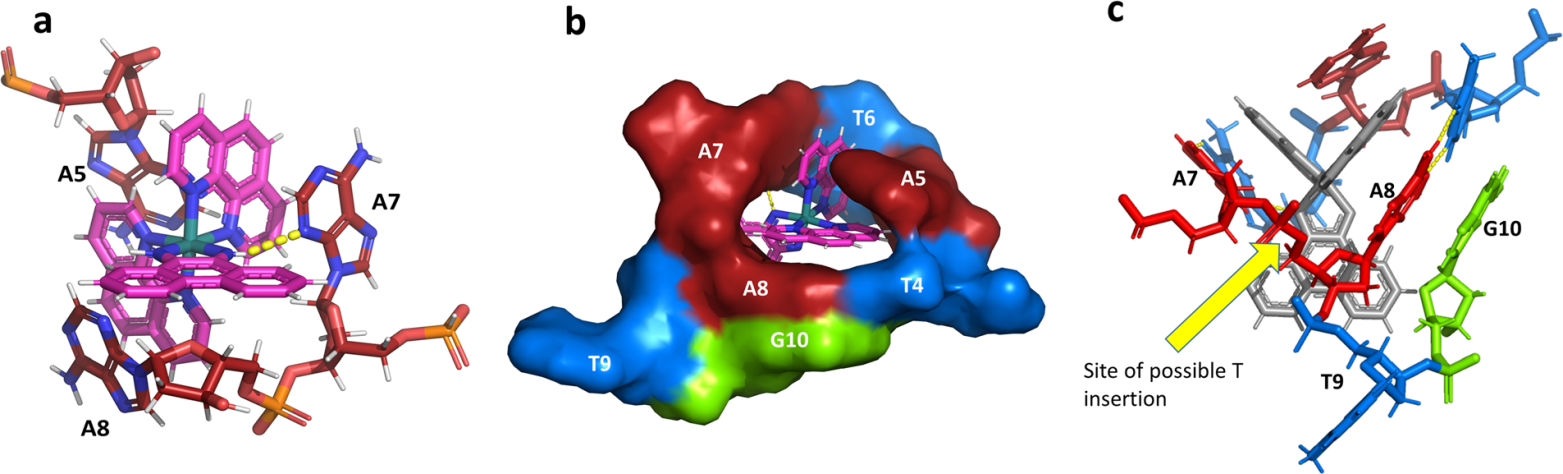
The environment of the Λ-complex. (a) The three adenine bases stacked on the two phenanthroline rings. View towards the phi ligand, which projects into the minor groove, to show the formation of a hydrogen bond (yellow dashes) between one imino H and N3 of A7. The angle formed between the rings of A7 and A8 is 80°; (b) the base pairs T4–A8 and T6–A7, with the stacked ‘bulge’ base A5 and the flipped out base T9, showed in surface mode to highlight the encapsulation of the Λ-complex. The view direction here is into the major groove, with the base G10 also included for clarity; (c) projection onto the phi ligand plane, to suggest how a binding mode similar to this could be possible at an ATA/TAT sequence, if a thymine base were present between A7 and A8.

This metal complex is bound exclusively to the central double mismatched section of this duplex, with the two bound complexes separated by a TA/TA step. The phen ligands sit on the major groove side of the duplex, such that the Ru atom is approximately equidistant to all three adenine bases, as shown in Fig. 4a. The closest approach is to N3 of A7. The binding mode has not been previously observed, and the adjacent residues A7 and A8 stack with the two Λ-phen ligands, one purine ring on each phen moiety. It could be described as a bulge binding site, with A5 the bulge. As the two phen moieties are approximately orthogonal, constrained by the rigid octahedral geometry of the metal centre, the extremely flexible DNA strand kinks such that the adenine base planes make an 80° dihedral angle, similar to the 86.5° angle between the phen ligands. The strength and specificity of the interaction is enhanced by the formation of a hydrogen bond between one imino H of the phi ligand and the N3 nitrogen of A7, with an N–N distance of 3.0 Å. The other imino H is bound to a water molecule, part of the extensive network of ordered water. From the opposing strand, the adenine ring of A5 also stacks with a phen ligand, making the plane approximately parallel with that of A8 on the first strand. Thus the metal complex is firmly and enantiospecifically bound, generating the kink, and is neither intercalated or inserted, but almost encapsulated, as shown in Fig. 4b. To our knowledge, such a binding mode, incorporated an adenosine bulge, has not been resolved before.

### The Δ-[Ru(phen)_2_phi]^2+^ binding site

**Fig. 5:**
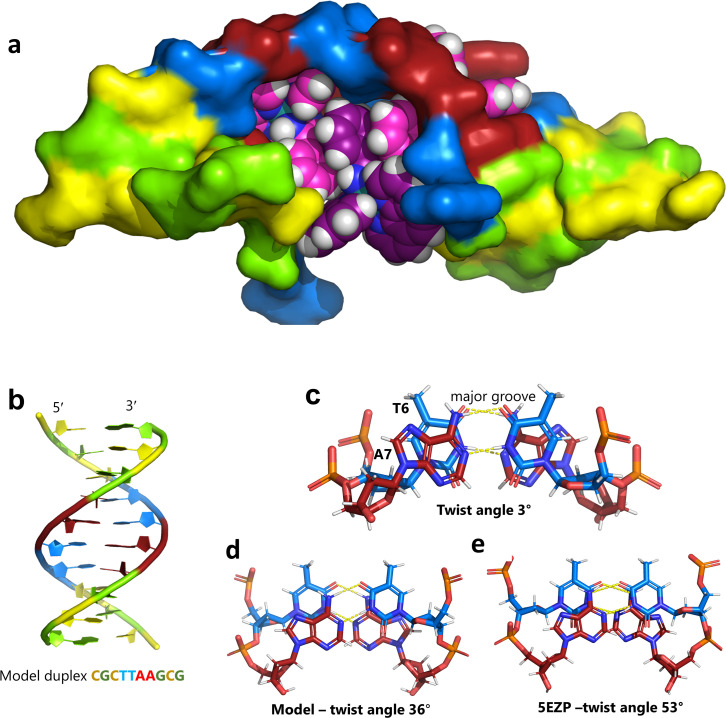
The effect of unwinding on the central TA/TA step. (a) The whole assembly with the nucleic acid component shown in surface mode and the metal complexes in spacefill mode; (b) the standard B-DNA model built using the parameters on the w3DNA web server; (c) projection of the TA/TA centre step in this work, showing the extensive pyrimidine-purine ring overlap which results from the unwinding from 36° to 3° (see Table 1); (d) similar projection of the TA/TA centre step in the model duplex, showing the lack of ring overlap; (e) similar projection from step 4 of the Pribnow box structure, PDB code 5EZP.

Unexpectedly, this enantiomer makes no direct nucleic acid contacts, as shown in Fig. 5a. The single complex lies on the twofold rotation axis, with the phi ligands of the Λ-complex stacking on the phi ligand of the Δ-enantiomer, and is enclosed by the kinked and metal bound duplex. The assembly of metal complexes has an overall +6 charge, so the principal interaction with the phosphate backbone is charge neutralisation, not stacking. A more detailed analysis of the rather limited environment of the delta complex is included in the discussion, as part of a consideration of the environment of the highly stacked TA/TA centre step.

### Data quality

A feature of the refined crystal structure is the high data quality (for complete statistics see the ESI†), which permits location of 69 fully occupied water positions with low temperature factors, a situation rarely encountered in nucleic acid crystallography. Most are clustered along the phosphate backbone, and the encapsulation of the ligands leaves little room for water approach, the only well-defined interaction being the hydrogen bond to one of the imino –NH groups on the phi ligand, as stated above.

### Nucleic acid conformation and the Pribnow box structure

**Fig. 6:**
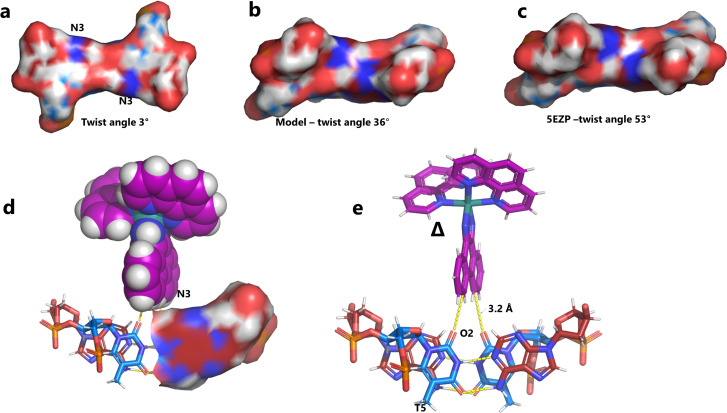
Comparison of TA/TA steps. (a) at the surface of the minor groove side of the central TA/TA step (same colour code) showing the contact surface for the Δ-complex in the crystal; (b) the corresponding surface for the model; (c) the corresponding surface (53° twist) in the Pribnow box structure, showing the much smaller cavity; (d) the Δ-complex (spacefill mode) with this surface, showing the hydrophobic interaction with the aromatic edge of the phi ligand of the complex, and only half the surface shown for clarity; (e) details of the interaction geometry shown in stick mode. Colour code used: thymine, blue; adenine, red; cytosine, yellow; and guanine, green.

There are five unique base pairs in this assembly, and an analysis of the conformation is included as Table S2.†Table 1 shows the local base step parameters, calculated using the program w3DNA,^34^ for the five unique steps in this structure. For comparison, the parameters for the model duplex d(CGCTTAAGCG)_2_ were calculated using the standard B-DNA fibre parameters, available on this server. The calculated twist and rise values are compared with the experimental one from this work. The CG/CG and GC/GC steps are close to normal, but the CT/AG and TA/TA steps show the much lower twist angles and decreased rise distances adjacent to the Λ-[Ru(phen)_2_phi]^2+^ binding site. This program cannot calculate meaningful base pair parameters for the TT/AA ‘step’ – so the most useful descriptor for the ‘step’ is probably the 80° angle between the *A* and *A* planes quoted above. Particularly striking is the almost parallel stacking of the central TA/TA step, which has the weakest stacking interaction of the ten standard base pair steps, and in other structures can show very high twist angles (overwinding) and a very small degree of base overlap (Fig. 6. Fig. 5b shows this standard B-DNA model, in which the effects of sequence dependence are included, so all the steps are already slightly different. Fig. 5c and d compare the standard model with the symmetric TA/TA central step in this work (the TA base pairs are related by the twofold axis of symmetry). Fig. 5e shows the even greater degree of unstacking in the Pribnow box well matched sequence at one of the TA/TA steps.^17^Table 1 also includes the corresponding parameters from the 1.65 Å Pribnow box structure, and the values for the third CT/AG step are similar, at around 20°. A duplex containing the Pribnow box consensus sequence eluded crystallisation for many years, but in 2016, using racemic DNA crystallography, crystals were obtained in four different space groups, of which three gave similar structures.^17^ The best statistics were obtained for PDB code 5EZF, in space group *Pbca*, so this is the comparison which may be most useful here. To obtain the duplex, the two strands d(CGCTATAATGCG) and d(CGCATATTAGCG) were cocrystallised, using strands of both left- and right-handed DNA. The crystals were assumed to be perfectly centrosymmetric for refinement purposes. These authors state that these models gave parameters in line with standard B-type DNA, but with some modifications to helical features. In the present work, the first three steps are comparable to those seen in the well-matched Pribnow box duplex, with the central TA/TA step very different. In the Pribnow box (5EZF) there are two TA/TA steps, both overwound and with complete base unstacking (Fig. 5d). This overwinding is also seen at the central intercalated TA/TA step of the Λ-[Ru(phen)_2_dppz]^2+^ bound duplex d(CCGGTACCGG)_2_, where the twist angle is 45°, and intercalation is from the minor groove.^35^ The structure here presents a complete contrast at this step (Fig. 5 and 6). Fig. 6 shows, using a surface representation, how this almost parallel stacking, normally so unfavourable, could facilitate the Δ-complex binding, in addition to the stacking between the complexes. Fig. 6a–c show the effect of increasing twist on the minor groove at this step. The 3° twist angle means that the minor groove shape is remarkably open (Fig. 6a) and creates the central cavity for the Δ-[Ru(phen)_2_phi]^2+^ ligand. The adenine base N3 atoms (blue) are only 4.5 Å apart in the standard model (Fig. 6b), and only 4.3 Å apart in 3EZF (Fig. 6c), but are 8.2 Å apart in this structure. This separation allows aromatic ring contacts are to the thymine carbonyl group 2-CO, which could introduce an element of polarity to the interaction, bearing in mind the 2+ overall charge of the complex. The closest approach distance is 3.2 Å, shown in Fig. 6d and e.

**Table 1:** Selected nucleic acid base step parameters (see also Table S2). Values included for comparison are italicised

Step	Shift (Å)	Slide (Å)	Rise (Å)	Rise (Å) (fibre)	Tilt (°)	Roll (°)	Twist (°)	Twist (°) (fibre)	Twist (°) 5EZF
CG/CG	0.27	0.45	3.52	*3.34*	3.4	7.1	32.5	*33.9*	*34.3*
GC/GC	0.72	−0.69	3.08	*3.38*	−2.8	−0.9	38.1	*38.0*	*42.1*
CT/AG	−0.94	−0.86	3.08	*3.35*	2.3	2.4	19.4	*34.7*	*22.6*
TT/AA	—	—	—	*3.36*	—	—	—	*35.9*	
TA/TA	0.00	2.66	2.58	*3.36*	0.0	4.8	2.8	*36.2*	*53.0*

### Intercalation, insertion and mismatch recognition

**Fig. 7:**
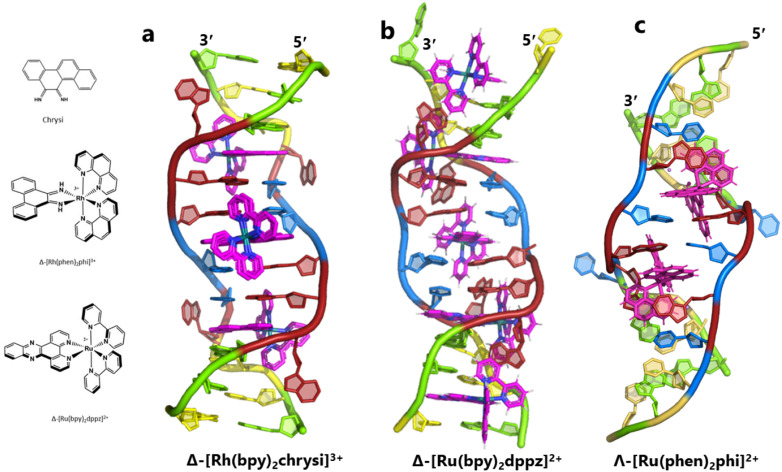
Intercalation, insertion, and re-pairing. (a) The mismatched sequence d(CGGAAATTACCG) crystallised with the complex Δ-[Rh(bpy)_2_chrysi]^3+^ (PDB code: 2O1I), where chrysi = 5,6-chrysene diimine; (b) the same sequence crystallised with Δ-[Ru(bpy)_2_dppz]^2+^; (c) the structure reported here, showing only the Λ-complex for clarity and ease of comparison.

The idea of recognising single mismatches by insertion and luminescence has been most well developed using the rhodium analogue of the complex used here, [Rh(phen)_2_phi]^3+^.^24^ An extended version of the phi ligand, chrysi, has been shown to recognise C–C and A–A mismatches by insertion from the DNA minor groove.^36^ The recognition is specific for delta enantiomers, and is driven by the stacking of the ejected bases, in a syn conformation, onto the phen ligands (in the context of intercalation, referred to as ‘ancillary ligands’), as shown in Fig. 7a.^37^ Here we see insertion from the minor groove and intercalation from the major groove, using the self-complementary d(CGGAAATTACCG) sequence. Insertion from the minor groove, at an A–A mismatch, is also known for Δ-[Ru(bpy)_2_dppz]^2+^ using the same sequence (Fig. 7b), but now, all the binding is from the minor groove.^38^ Here also, the binding mode is enantiospecific for the delta enantiomer. These binding modes do not cause overall curving or kinking of the helix. In contrast, the Λ-[Ru(phen)_2_phi]^2+^ complex used in this work neither intercalates nor inserts, but recognises the ‘bulge’ at A5 and binds from the major groove with stacking of the bulged adenine as well as the two base-paired adenines to create a kink whose angle is primarily determined by the approximately octahedral geometry at Ru to give an approximately 80° kinking. As there are two such sites, overall the helix is bent, as shown in Fig. 7c, and the phen ligands are here not ancillary, but the main drivers of the interaction, with the phi ligand interacting only by hydrogen bond formation to the already base paired A7. The common feature linking insertion and what could be called the bulge recognition seen here is the role of the adenine base stacking on the phen ligands of each metal complex, possibly related to the absence of a polarising carbonyl group in adenine. Bulge recognition using Δ-[Rh(bpy)_2_(chrysi)]^3+^ has been explicitly studied for single base bulges.^39,40^ These authors assessed the thermodynamics of bulge binding and the specific DNA cleavage made possible by such recognition, which they suggest is by a similar mechanism as that seen for the insertion mode shown in Fig. 7a, from the minor groove. They highlight the enantiospecificity of recognition, which suggests that the binding mode might have some similarity to that seen here, and they also point out that DNA bending is known to be a feature of bulged sites. The crystal structures of the d(GCGAAGC) and d(GCGAAAGC) duplexes also show bending, here at double and triple mismatched sites.^41^

## Conclusions

The structure reported here shows, with excellent quality data, a new binding mode for Λ-[Ru(phen)_2_phi]^2+^ which is in addition to the three binding modes recently described by us.^6^ In that work, we saw symmetrical major groove intercalation at a TA/TA step, angled minor groove intercalation at a GG/CC step, and linking of decamer duplexes at the terminal CC/GG step to give an orthogonal arrangement of intercalated duplexes in the final assembly. The closest parallel to the binding mode seen here is that seen at the linking of the duplexes, where in both cases the overall angle was determined by the approximately octahedral geometry of the complex. The use of transition metal complexes as DNA binding agents gives rise to features such as this, with no real parallel among purely organic binders.

One broader aim of this study was to examine the behaviour of Λ-[Ru(phen)_2_phi]^2+^ with AT-rich DNA sequences, but crystals have not been obtained to date with the well-matched sequences. As outlined in the Introduction, the annealed duplex, d(GCTTTATAAAGC)_2_, gives a +22.4 °C increase in UV thermal melting temperature compared to the untreated control (Δ*T*_m_) with Λ-[Ru(phen)_2_phi]^2+^, but only +5.8 °C with Δ-[Ru(phen)_2_phi]^2+^.^6^ The enantiospecific stabilisation of this sequence, and the uracil analogue d(GCUUUAUAAAGC)_2_, could be due to a binding mode which was not simple intercalation, but perhaps a version of the re-pairing and bulge binding seen here, which is a binding mode stabilised by strong stacking interactions and a hydrogen bond formation.

This structure suggests that Λ-[Ru(phen)_2_phi]^2+^ has more favourable major groove binding properties than the Δ enantiomer, leading to thermodynamically favourable intercalation or insertion interactions. Meanwhile the Δ complex affords limited *T*_m_ stabilisation, and is not directly bound in this study. Further structural and solution studies of DNA duplexes with the enantiomers of [Ru(phen)_2_phi]^2+^ are in progress, with the specific aim of determining what would be another sequence specific binding mode of this complex.

## Data availability

The structure has been deposited in the PDB with code 8CMM.

## Author contributions

TDPO (conceptualisation, investigation, writing), KMcQ (data curation, methodology, validation, supervision) NP (data acquisition), DJC (supervision, resources), AK (conceptualisation, funding acquisition, project administration, writing), CJC (conceptualisation, funding acquisition, supervision, visualisation, writing).

## Conflicts of interest

There are no conflicts to declare.

## Supplementary Material

SC-015-D4SC01448K-s001

SC-015-D4SC01448K-s002
