# Correction: Re-pairing DNA: binding of a ruthenium phi complex to a double mismatch

**DOI:** 10.1039/d4sc90174f

**Published:** 2024-09-06

**Authors:** Tayler D. Prieto Otoya, Kane T. McQuaid, Neil G. Paterson, David J. Cardin, Andrew Kellett, Christine J. Cardin

**Affiliations:** a Department of Chemistry, University of Reading Whiteknights, Reading RG6 6AD UK c.j.cardin@reading.ac.uk; b Diamond Light Source Ltd, Harwell Science and Innovation Campus Didcot Oxfordshire OX11 0DE UK; c SSPC, The Science Foundation Ireland Research Centre for Pharmaceuticals, School of Chemical Sciences, Dublin City University Glasnevin, Dublin 9 Ireland

## Abstract

Correction for ‘Re-pairing DNA: binding of a ruthenium phi complex to a double mismatch’ by Tayler D. Prieto Otoya *et al.*, *Chem. Sci.*, 2024, **15**, 9096–9103, https://doi.org/10.1039/D4SC01448K.

The authors regret that in the published version of the paper, the letters in Fig. 2b have shifted upwards on the left-hand side. The correct version of Fig. 2, with its caption, is given herein.

**Fig. 2:**
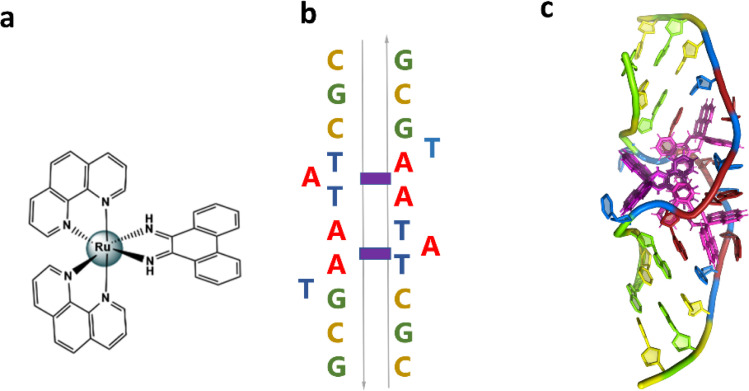
(a) Structural formula of Λ-[Ru(phen)_2_phi]^2+^; (b) schematic showing the re-pairing of the bases in the reported structure. The purple blocks highlight the binding sites of the complex. (c) Image showing the large DNA bending. The overall assembly, characterised by a twofold rotational symmetry. Each asymmetric unit is made up of a DNA single strand binding a Λ-[Ru(phen)_2_phi]^2+^ with occupancy 1 and a Δ-[Ru(phen)_2_phi]^2+^ with occupancy 0.5. The ruthenium complexes are shown in purple.

The Royal Society of Chemistry apologises for these errors and any consequent inconvenience to authors and readers.

