# Supplementary material for: Re-pairing DNA: binding of a ruthenium phi complex to a double mismatch
Source: Chem Sci. 2024 May 16;15(24):9096–103. doi: 10.1039/d4sc01448k (PMC11186304; doi:10.1039/d4sc01448k)
Supplement: SC-015-D4SC01448K-s001 [file SC-015-D4SC01448K-s001.pdf]

Electronic Supplementary Material (ESI) for Chemical Science.
This journal is © The Royal Society of Chemistry 2024

Re-pairing DNA: Binding of a ruthenium phi complex to a double mismatch

Tayler D. Prieto Otoya[a], Kane McQuaid[a], Neil G. Paterson[b], David Cardin[a], Andrew Kellett[c], Christine J. Cardin[a].

[a]Department of Chemistry, University of Reading, Whiteknights, Reading, RG6 6AD;

[b]Diamond Light Source Ltd., Harwell Science and Innovation Campus, Didcot, Oxfordshire, OX11 0DE, UK

[c]SSPC, The Science Foundation Ireland Research centre for Pharmaceuticals, School of Chemical Sciences, Dublin City University, Glasnevin, Dublin 9, Ireland.

* To whom correspondence should be addressed; Email: c.j.cardin@reading.ac.uk

Figure S1 – Perspective view of the unit cell from the crystal structure of *rac*-[Ru(phen)$_2$phi]$^{2+}$ with DNA mismatch, illustrating the solvent channels.

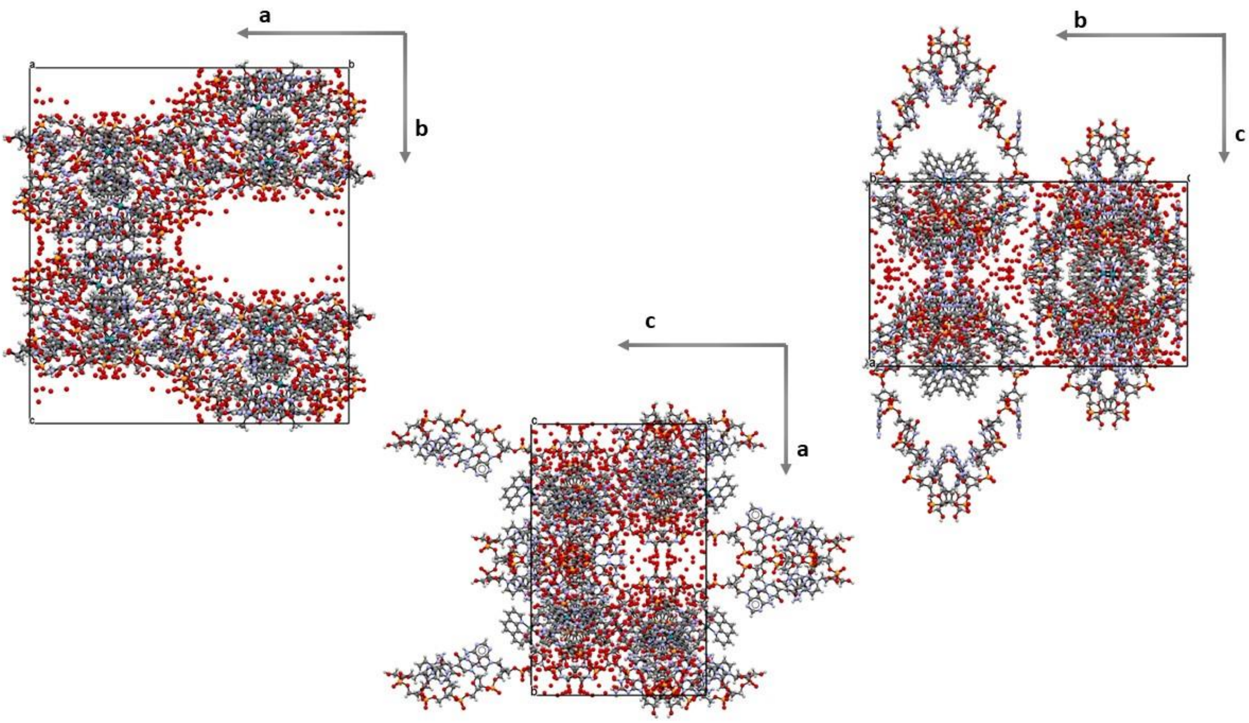

Figure S2 – Electron density around a) [Ru(phen)2phi]2+, and b) guanine-cytosine base pair, showing the high-quality data of this crystal structure. Map contouring level 0.8800 e/Å^3 (1.5σ).

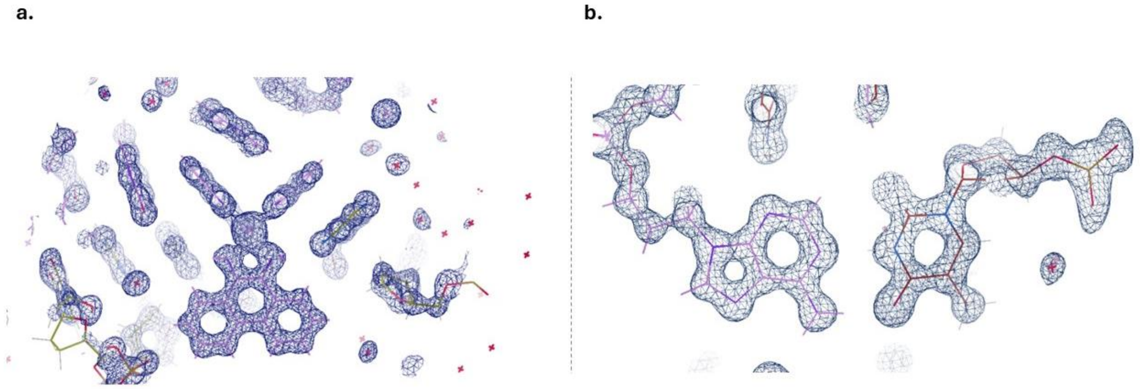

Figure S3. The location of 69 water molecules (red spheres) in this structure (8CMM), shown from both sides of the DNA backbone. The asymmetric unit, shown as spheres. Nucleic acid component – grey; ruthenium complexes – pink. K^{+} and Li+ shown as small spheres for clarity.

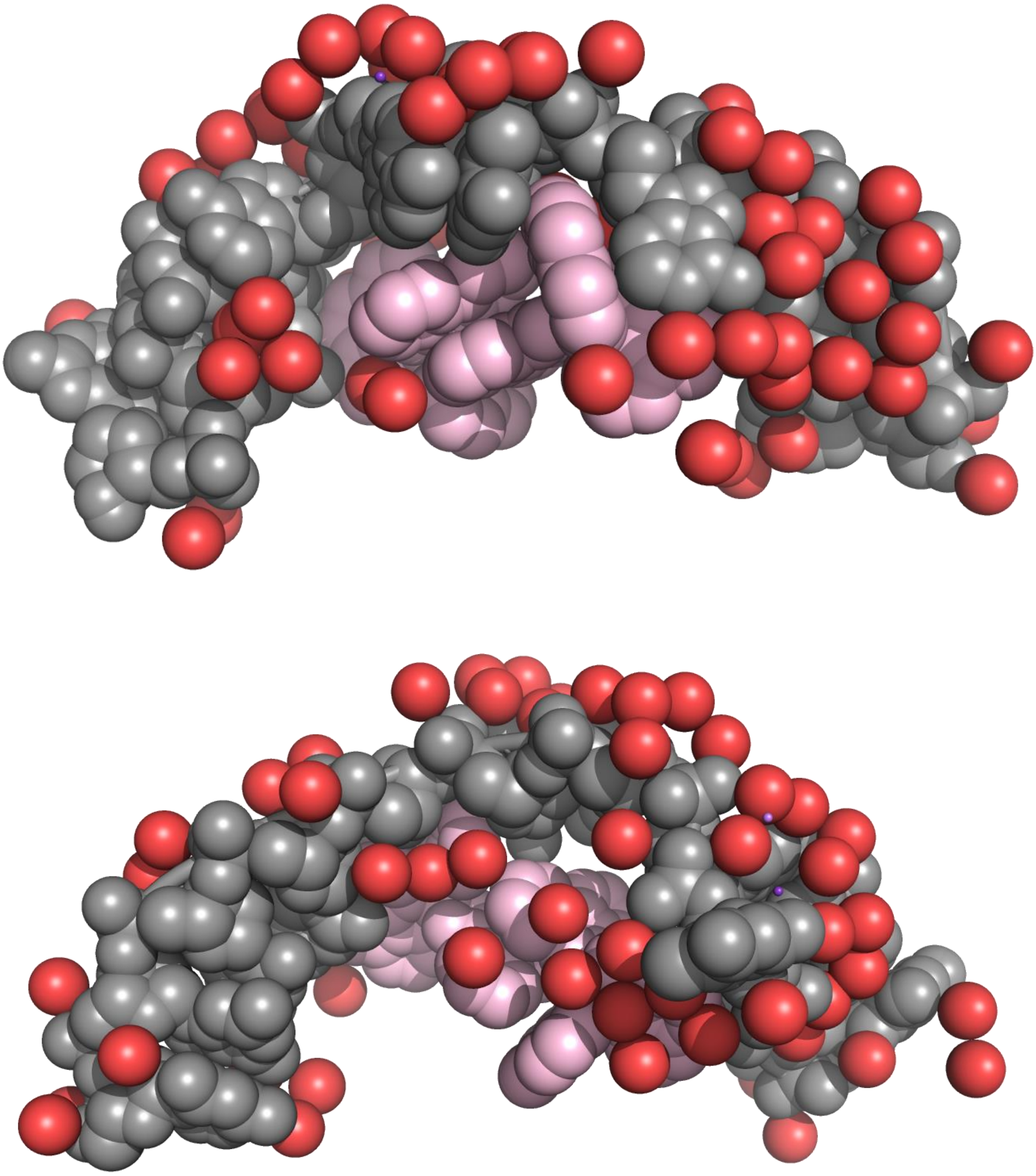

Table S1 – Summary, data collection and refinement statistics.

**Data collection**	
Space group	$I2_12_12_1$
Resolution (Å)	36.20 – 0.90
Data collection temperature (K)	100
R_{merge}	0.044
R_{pim}	0.013
R_{meas}	0.046
Anisotropy	0.492
F_o, F_c correlation	0.98
I/σI	1.00 (at 0.90 Å)
$CC_{1/2}$	0.999
Completeness for range %	92.47
Data redundancy	12.1
Wilson B factor (Å^2)	10.08
Total number of atoms	447
Average B, all atoms (Å^2)	13.0

**Refinement**	
N_o reflections measured	27375
R_{work}/R_{free}	0.1359/0.1594
Data cutoff (sigma(F))	NONE
Free R value test set count	1425
Mean B value (overall A^2)	11.224
**PDB ID**	**8CMM**

Table S2. Conformational analysis- The list of parameters given below correspond to the 5´ to the 3´ direction of strand I, and 3´ to 5´ direction of strand II.

Local base-pair parameters

bp		Shear	Stretch	Stagger	Buckle	Propeller	Opening
1	C-G	0.35	-0.21	0.18	7.69	-15.86	-0.85
2	G-C	0.03	-0.15	0.01	3.21	-18.73	2.98
3	C-G	0.21	-0.16	0.09	3.42	-10.57	-1.01
4	T-A	-0.16	-0.09	-0.32	13.17	-5.09	4.75
5	T-A	0.05	-0.17	0.26	-18.22	15.64	-1.79
6	A-T	-0.05	-0.17	0.26	18.22	15.64	-1.79
7	A-T	0.16	-0.09	-0.32	-13.17	-5.09	4.75
8	G-C	-0.21	-0.16	0.09	-3.42	-10.57	-1.01
9	C-G	-0.03	-0.15	0.01	-3.21	-18.73	2.98
10	G-C	-0.35	-0.21	0.18	-7.69	-15.86	-0.85
11	G-G	-0.00	-8.41	2.09	0.00	-33.00	-157.99
12	C-G	0.35	-0.21	0.18	7.69	-15.86	-0.85
13	G-C	0.03	-0.15	0.01	3.21	-18.73	2.98
14	C-G	0.21	-0.16	0.09	3.42	-10.57	-1.01
15	T-A	-0.16	-0.09	-0.32	13.17	-5.09	4.75
16	T-A	0.05	-0.17	0.26	-18.22	15.64	-1.79
17	A-T	-0.05	-0.17	0.26	18.22	15.64	-1.79
18	A-T	0.16	-0.09	-0.32	-13.17	-5.09	4.75
19	G-C	-0.21	-0.16	0.09	-3.42	-10.57	-1.01
20	C-G	-0.03	-0.15	0.01	-3.21	-18.73	2.98
21	G-C	-0.35	-0.21	0.18	-7.69	-15.86	-0.85
	ave.	0.00	-0.55	0.14	-0.00	-8.17	-6.75
	s.d.	0.20	1.80	0.49	10.83	13.47	34.75

Local base-pair step parameters

step	Shift	Slide	Rise	Tilt	Roll	Twist
1 CG/CG	0.27	0.45	3.52	3.40	7.19	32.54
2 GC/GC	0.72	-0.69	3.23	-2.86	-0.91	38.15
3 CT/AG	-0.94	-0.86	3.08	2.33	2.45	19.43
4 TT/AA	----	----	----	----	----	----
5 TA/TA	-0.00	2.66	2.58	-0.00	4.83	2.86
6 AA/TT	----	----	----	----	----	----
7 AG/CT	0.94	-0.86	3.08	-2.33	2.45	19.43
8 GC/GC	-0.72	-0.69	3.23	2.86	-0.91	38.15
9 CG/CG	-0.27	0.45	3.52	-3.40	7.19	32.54
10 GG/GC	----	----	----	----	----	----
11 GC/GG	----	----	----	----	----	----
12 CG/CG	0.27	0.45	3.52	3.40	7.19	32.54
13 GC/GC	0.72	-0.69	3.23	-2.86	-0.91	38.15
14 CT/AG	-0.94	-0.86	3.08	2.33	2.45	19.43
15 TT/AA	----	----	----	----	----	----
16 TA/TA	0.00	2.66	2.58	-0.00	4.83	2.86
17 AA/TT	----	----	----	----	----	----
18 AG/CT	0.94	-0.86	3.08	-2.33	2.45	19.43
19 GC/GC	-0.72	-0.69	3.23	2.86	-0.91	38.15
20 CG/CG	-0.27	0.45	3.52	-3.40	7.19	32.54
ave.	0.00	0.07	3.18	-0.00	3.18	26.16
s.d.	0.68	1.23	0.31	2.78	3.27	12.42

Local base-pair helical parameters

step	X-disp	Y-disp	h-Rise	Incl.	Tip	h-Twist
1 CG/CG	-0.51	0.14	3.55	12.60	-5.95	33.47
2 GC/GC	-0.94	-1.45	3.19	-1.39	4.36	38.26
3 CT/AG	-3.58	3.77	2.81	7.18	-6.85	19.72
4 TT/AA	----	----	----	----	----	----
5 TA/TA	-8.79	-0.00	3.60	59.34	0.00	5.61
6 AA/TT	----	----	----	----	----	----
7 AG/CT	-3.58	-3.77	2.81	7.18	6.85	19.72
8 GC/GC	-0.94	1.45	3.19	-1.39	-4.36	38.26
9 CG/CG	-0.51	-0.14	3.55	12.60	5.95	33.47
10 GG/GC	----	----	----	----	----	----
11 GC/GG	----	----	----	----	----	----
12 CG/CG	-0.51	0.14	3.55	12.60	-5.95	33.47
13 GC/GC	-0.94	-1.45	3.19	-1.39	4.36	38.26
14 CT/AG	-3.58	3.77	2.81	7.18	-6.85	19.72
15 TT/AA	----	----	----	----	----	----
16 TA/TA	-8.79	-0.00	3.60	59.34	0.00	5.61
17 AA/TT	----	----	----	----	----	----
18 AG/CT	-3.58	-3.77	2.81	7.18	6.85	19.72
19 GC/GC	-0.94	1.45	3.19	-1.39	-4.36	38.26
20 CG/CG	-0.51	-0.14	3.55	12.60	5.95	33.47
ave.	-2.69	-0.00	3.24	13.73	0.00	26.93
s.d.	2.89	2.24	0.33	20.10	5.58	11.77
